# Real-Time Analysis of SARS-CoV-2-Induced Cytolysis Reveals Distinct Variant-Specific Replication Profiles

**DOI:** 10.3390/v15091937

**Published:** 2023-09-16

**Authors:** Sarah E. Scheuermann, Kelly Goff, Lori A. Rowe, Brandon J. Beddingfield, Nicholas J. Maness

**Affiliations:** 1 Division of Microbiology, Tulane National Primate Research Center, Covington, LA 70433, USA; sspence3@tulane.edu (S.E.S.); kgoff@tulane.edu (K.G.); lrowe1@tulane.edu (L.A.R.); 2 Department of Microbiology and Immunology, Tulane University School of Medicine, New Orleans, LA 70112, USA

**Keywords:** SARS-CoV-2 variants, real-time cell analysis, viral replication

## Abstract

The ability of each new SARS-CoV-2 variant to evade host humoral immunity is the focus of intense research. Each variant may also harbor unique replication capabilities relevant for disease and transmission. Here, we demonstrate a new approach to assessing viral replication kinetics using real-time cell analysis (RTCA). Virus-induced cell death is measured in real time as changes in electrical impedance through cell monolayers while images are acquired at defined intervals via an onboard microscope and camera. Using this system, we quantified replication kinetics of five clinically important viral variants: WA1/2020 (ancestral), Delta, and Omicron subvariants BA.1, BA.4, and BA.5. Multiple measures proved useful in variant replication comparisons, including the elapsed time to, and the slope at, the maximum rate of cell death. Important findings include significantly weaker replication kinetics of BA.1 by all measures, while BA.5 harbored replication kinetics at or near ancestral levels, suggesting evolution to regain replicative capacity, and both an altered profile of cell killing and enhanced fusogenicity of the Delta variant. Together, these data show that RTCA is a robust method to assess replicative capacity of any given SARS-CoV-2 variant rapidly and quantitatively, which may be useful in assessment of newly emerging variants.

## 1. Introduction

The recently emerged coronavirus, SARS-CoV-2, has been responsible for an ongoing pandemic since 2019. Since its initial emergence, it has continuously evolved, fueled by immune pressure from vaccination and infection in the human population. From the early widely circulating strain, deemed WA1/2020 in the United States [1], important variants have included the first D614G mutant [2,3], Alpha (B.1.1.7) [4,5,6], Beta (B.1.351) [7,8], Gamma (P.1) [9,10], Delta (B.1.617.2) [11,12,13], Lambda (C.37) [14], Mu (B.1.621) [15], and others. Since it was first detected in November of 2021, the variant of concern, Omicron, has dominated the global pandemic and has since spawned multiple sublineages, many of which show substantial variation relative to the original Omicron variant from which they evolved [16,17]. New variants/subvariants are routinely discovered, such as Omicron XBB.1.5 [18] and BQ.1.1 [19], due to immune evasion and other selective pressures. These subvariants now define the pandemic.

New variants rapidly spread through populations and often become dominant before declining in frequency and being replaced by a new variant, often with even greater antibody escape capacity [11,12,17,20,21]. This has resulted in monoclonal antibody therapeutics and infection-derived antibodies becoming obsolete as the pandemic has continued, due to the highly specific nature of antibody binding [19,22,23,24,25]. Vaccines are affected as well, though possibly to a lesser degree [7,16,26,27,28,29,30]. Immunity generated by the widely used mRNA vaccines also shows decreased neutralizing capacity for variants relative to the ancestral variant, with serum from vaccinees having up to threefold decreased neutralization for the Delta variant [31] and far greater reductions in neutralization of Omicron and its subvariants. Variants may evade cellular immunity as well [32], though this appears to be less widespread than evasion of humoral immunity.

In addition to immune escape, mutations in newly emerged variants may also impact viral infection, replication, and transmission. Mutations within the receptor binding domain (RBD) of the spike protein may modify binding and uptake of virus, while spike mutations outside the RBD may alter aspects of replication in other ways favorable for intrahost viral dynamics. Altered replication dynamics may also impact disease severity and interhost transmission [3,20,33]. In prior work, Omicron showed reduced viral replication kinetics in cell culture, potentially due to a reduced ability to antagonize the interferon response as compared to Delta [34], but this comparison is complex as Omicron actually shows greater replication than Delta in nasal cells and bronchi explants, while the opposite is true in lung explants and lung cells [35,36,37,38]. These variants show differential reliance on TMPRSS2 for entry [39], with Omicron much less on TMPRSS2 for cell entry, which likely explains cell-type-dependent differential replication kinetics. Which of these variables explain the reduced severity of disease associated with the Omicron variant is not known. Importantly, mutations underlying these altered kinetics may also lie outside of spike in structural or nonstructural proteins [40]. Altogether, these findings highlight the importance of robust characterization of viral kinetics in live, whole virus assays.

A relatively new technology that has expanded into virology laboratories in recent years, termed real-time cell analysis (RTCA) using the Agilent xCELLigence eSight system, has the potential to rapidly assess viral replication kinetics and other important parameters. This technology is based upon real-time measurements of electrical impedance of a cell monolayer [41,42,43]. The impedance is a correlate of monolayer integrity, with impedance falling as cells are destroyed, including as a result of lytic viral infection. This is reflected in a unitless value termed cell index, which can be monitored on a per-well basis over the course of multiple days and combined with visual monitoring of the monolayer using an integrated microscope and camera. We herein describe the use of this technology as a platform for detailed examination of the in vitro kinetics of replication of multiple SARS-CoV-2 variants of concern. We show RTCA to be an ideal tool for viral characterization that can aid in the elucidation of unique aspects of emerging variants during a rapidly evolving pandemic.

## 2. Materials and Methods

### 2.1. Virus and Cells

Multiple SARS-CoV-2 variants were used to infect Vero-TMPRSS2 cells (# JCRB1819, JCRB Cell Bank, Osaka, Japan). Cells were cultured in Dulbecco’s Modified Eagle’s Medium (DMEM) supplemented with 10% fetal bovine serum (FBS) and 1% antibiotic–antimycotic additionally supplemented with 2% G418 Sulfate Solution.

The following viruses were received from BEI Resources: NR-54001, icSARS-CoV-2-WT (WA1/2020); hCoV-19/USA/MD-HP20874/2021 WCCM (Omicron BA.1): NR-58620, hCoV-19/USA/COR-22-063113/2022 WCCM (Omicron BA.5); and NR-56806, hCoV-19/USA/MD-HP30386/2022 WCCM (Omicron BA.4); NR-55672, hCoV-19/USA/MD-HP05647/2021 (Delta B.1.617.2). Virus was propagated in Vero-TMPRSS2 cells to create stocks. Sequences of new stocks were confirmed by Illumina sequencing as previously described [44]. Genome assembly and variant analysis was performed using the DRAGEN COVID Lineage pipeline as an Illumina BaseSpace App following standard protocol, except for a custom primer BED file containing the SWIFT primers.

### 2.2. TCID_50_

Median tissue culture infectious dose (TCID_50_) was performed on each stock to quantify the amount of active, replication-competent virus. Vero TMPRSS2 cells were plated in 48-well tissue culture treated plates to be subconfluent at time of assay. Cells were washed with serum-free DMEM and 50 µL of virus was allowed to adsorb onto the cells for 1 h at 37 °C and 5% CO_2_. After adsorption, cells were overlayed with DMEM containing 2% FBS and 1% antibiotic-–antimycotic (#15240062, Thermo Scientific, Waltham, MA, USA). Plates were incubated for 7–10 days before being observed for cytopathic effect (CPE). Any CPE observed relative to control wells was considered positive and used to calculate TCID_50_ by the Reed and Muench method [45].

### 2.3. Real-Time Cell Analysis Assay Setup and Data Analysis

The real-time cell analysis assay is performed by infecting monolayers of target cells that are plated in specialized 96-well plates with embedded electrodes. The plates are placed on cradles of the instrument, which measures electrical impedance through cell monolayers and produces an arbitrary value termed cell index (see Figure S1 for graphed values of cell index over time). These data can be analyzed in a variety of ways, including calculating and comparing the time it takes to reach the maximum rate of drop of the cell index (the maximum rate of cell death), area under the curve, and others. Cell index values are obtained every 15 minutes, while microscopic real-time images are obtained of each well every hour for the entirety of the assay. Specifically, Vero-TMPRSS2 cells were plated in 96-well tissue culture-treated E-Plate VIEW plates (#300-601-020, Agilent, Santa Clara, CA, USA) to be subconfluent at time of assay initiation. Viral strains were each diluted with DMEM containing 2% FBS and 1% antibiotic–antimycotic to the same starting concentration of 1 × 10^5^ TCID_50_ followed by 1:10 serial dilutions for a total of seven dilutions. Media were removed from the wells of the 96-well plate, and 100 µL of virus samples were added. A total of 100 µL of 2% FBS 1% antibiotic–antimycotic DMEM were added to the negative control wells. The plates were then placed in the incubator at 37 °C, 5% CO_2_, on the xCELLigence RTCA eSight impedance and imaging cradles (cradles 1–3). The plate layouts and experiment schedule were defined in the eSight software v.1.1.1. Impedance measurements for each well were collected every 15 min and images for each well collected every 60 min over the course of 5 days. Six replicate wells were used for each experimental condition.

Cell index values over time were graphed in the xCELLigence software v.1.1.1. Graphs for all plates were then normalized at the same timepoint (11.76 h) with the delta cell index function to add a constant to the cell index of each well. Area under the curve (AUC) for each replicate was calculated using the area under the curve analysis function in the Prism software v.9.5.0. The AUC baseline parameters were set based on the lowest delta cell index (impedance) value for each replicate. The Kruskal–Wallis test was then performed to compare the total AUC of variants at each concentration.

Slope values over time were graphed in the xCELLigence software v.1.1.1 and normalized to the same timepoint (11.76 h) prior to exporting data. The lowest slope (steepest downward slope) value of each replicate was identified as the “max slope”. The time at which each replicate reached the max slope was also recorded. The Kruskal–Wallis test was then performed to compare the value and time of max slopes of each variant at each concentration.

### 2.4. Viral Nucleoprotein Quantification Assay

Tissue-culture-treated 96-well plates (#CC762-7596, USA Scientific, Ocala, FL, USA) were seeded with Vero TMPRSS2 cells to be subconfluent at time of assay. Viral strains were each diluted with DMEM containing 2% FBS and 1% antibiotic–antimycotic to the same starting concentration of 1 × 10^5^ TCID_50_ followed by 1:10 serial dilutions for a total of five dilutions. Media were removed from the wells of the 96-well plate, and 100 µL of virus samples were added. A total of 100 µL of 2% FBS 1% antibiotic–antimycotic DMEM were added to the negative control wells. Plates were then incubated at 37 °C, 5% CO_2_, and each plate was formalin fixed at a specific timepoint from 2 to 72 h post viral inoculation. Plates were stained for SARS-CoV/SARS-CoV-2 Nucleocapsid following the staining protocol outlined in a previous report [46] using SARS-CoV/SARS-CoV-2 Nucleocapsid Antibody, Mouse Mab (#40143-MM08, Sino Biological, Beijing, China), and Goat anti-Mouse IgG (H + L) Cross-Adsorbed Secondary Antibody, HRP (#A16072, Invitrogen, Waltham, MA, USA). Wells were developed with 1-Step Ultra TMB-ELISA (#34028, Thermo Scientific, Waltham, MA, USA), and the optical density at 450 nm (OD_450_) was measured using a Tecan Sunrise microplate reader. The OD_450_ values for the negative control wells were averaged and subtracted from each sample replicate to account for signal background. OD_450_ values were then graphed using Prism software v.9.5.0.

## 3. Results

### 3.1. Cell Index Patterns Differ between Variants

Vero/TMPRSS2 cells were inoculated with different SARS-CoV-2 variants of concern to characterize the viral ability to infect and destroy the monolayer, by near-continuous monitoring of the monolayer impedance to electricity as a correlate of cellular death. Readings of the monolayer impedance were taken every fifteen minutes over the course of five days in order to generate impedance curves (Figure S1). Various characteristics of these curves were analyzed, including time to maximum (max) slope, value of max slope, and area under the curve (AUC) of the impedance drop. These were performed using multiple dilutions of virus in order to characterize differences at a range of infectious doses. The specifics of these patterns and their differences by variant are captured in this manner.

### 3.2. Slope Characteristics Differ between Variants

**Figure 1:**
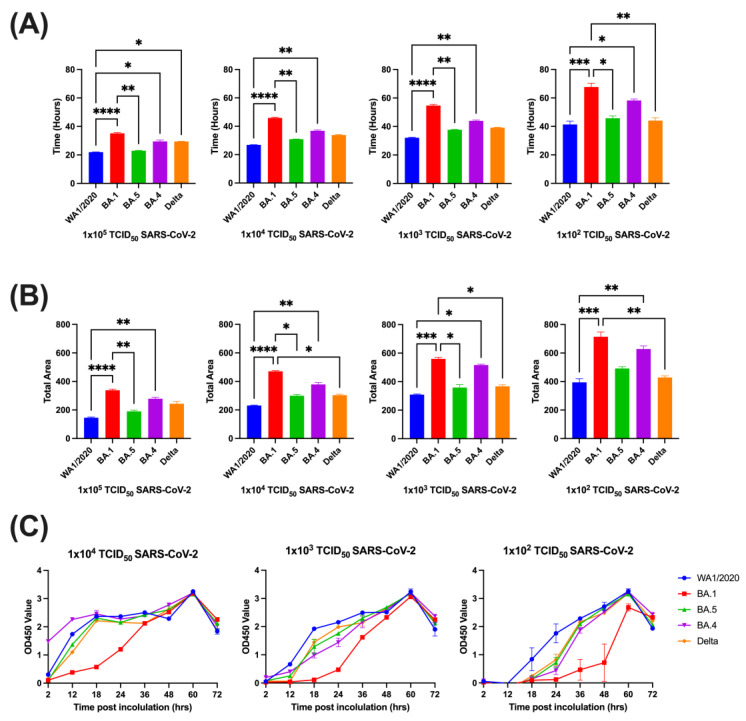
Kinetic comparisons of Vero-TMPRSS2 cells inoculated with multiple SARS-CoV-2 variants of concern. Time to reach max slope of inoculated cells (**A**) and area under the curve (AUC) (**B**) of cell index. Data are represented as mean with SEM. Groups were compared via Kruskal–Wallis test (*, *p* < 0.05; **, *p* < 0.01; ***, *p* < 0.001; ****, *p* < 0.0001). Data were collected from 6 replicate wells for each condition. The optical density value at 450 nm of each variant across multiple viral concentrations over time is represented as means with SEM (**C**).

The time taken to reach the max slope was the first metric used to assess replication kinetics of the different variants across a range of dilutions. In all dilutions, WA1/2020 reached max slope more quickly than all other variants, save for Delta at 1 × 10^4^ and 1 × 10^3^ TCID_50_, though the trend persisted with those comparisons as well. BA.1 was the slowest at reaching max slope across dilutions, though not all pairwise comparisons reached statistical significance. BA.4, BA.5, and Delta were not significantly different from each other across dilutions (Figure 1A).

The area under the cell index curve (AUC) was next analyzed for each variant and dilution. WA1/2020 had the lowest AUC across all dilutions, being significantly lower than BA.1, BA.4, and BA.5 at various dilutions, but never reaching significance compared to Delta. BA.1 had the highest AUC of the variants at all dilutions, being significantly higher than Delta at three of four dilutions and higher than WA1/2020 at all dilutions (Figure 1B). To validate these findings in the context of a more traditional assay that directly measures viral replication, we used a nearly identical assay design but with direct quantitative labeling of the viral nucleocapsid protein as the readout, and with assay sampling at several time points after infection. Data from this assay were similar to those produced using our novel impedance-based assay, particularly with regards to the observation that BA.1 showed substantially slower viral replication. However, this assay showed minimal to no differences across the other variants, but the ancestral WA1/2020 showed the most rapid viral kinetics (Figure 1C).

**Figure 2:**
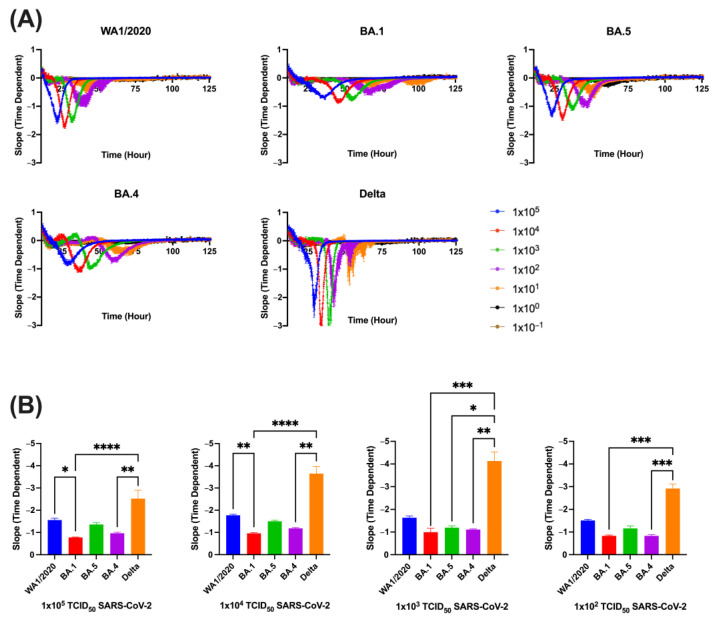
Slope of Vero/TMPRSS2 monolayers inoculated with SARS-CoV-2 variants. The value of each variant’s slope across multiple viral concentrations over time is represented as means with SEM. Legend indicates quantity of virus in TCID50 added onto cell monolayers (**A**). The value of each variant’s max slope across multiple viral concentrations graphed as averages with SEM (**B**). Groups were compared via Kruskal–Wallis test (*, *p* < 0.05; **, *p* < 0.01; ***, *p* < 0.001; ****, *p* < 0.0001). Data were collected from 6 replicate wells for each experimental condition.

Since tracking of cell index over time is a measure of cell death over time, the slope of the rate of cell death can also be tracked and assessed for variant-specific differences. Specifically, the absolute value of the slope generated by each variant was assessed. A clear pattern emerged wherein Delta exhibited the steepest absolute slope while BA.1 exhibited the least steep slope (Figure 2A). Statistical analyses validated these observations. Delta generated the steepest slope of all variants across all dilutions, being significantly different than BA.1, BA.4, and BA.5 at some dilutions, though it never reached significance compared to WA1/2020 due to a slightly higher standard error. BA.1 had the least steep slope at all dilutions. At the two highest viral inputs, 1 × 10^5^ and 1 × 10^4^ TCID_50_, WA1/2020 had a steeper slope than BA.1, though the trend did not continue for the lower dilutions (Figure 2B).

### 3.3. AUC Relationships Correlate with Slope Characteristics and Concentration of Viral Inoculum

**Figure 3:**
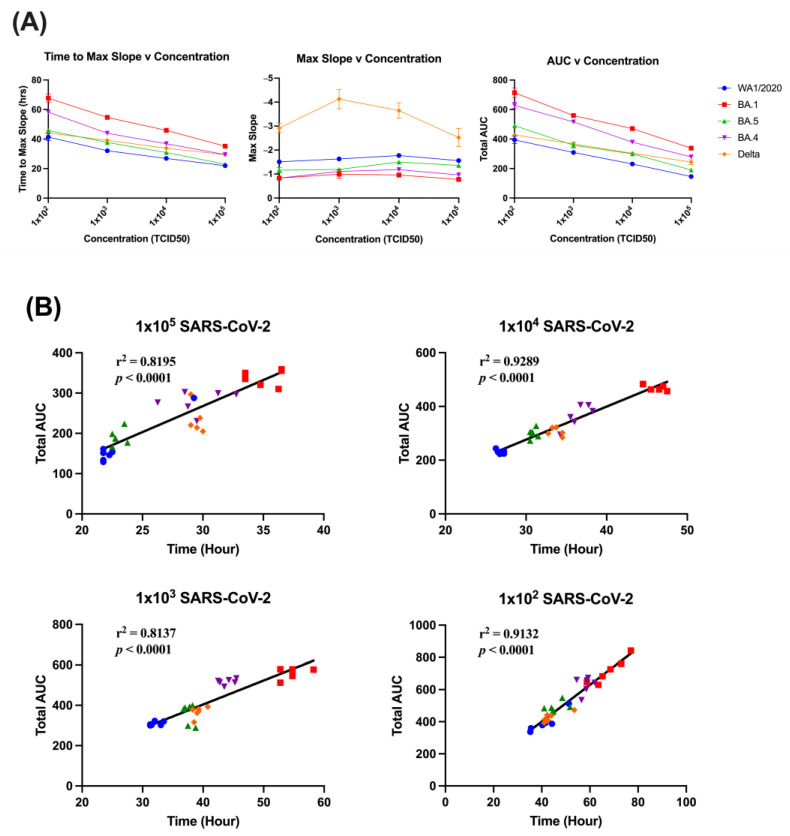
Slope and AUC relationships of SARS-CoV-2 variants. Relationships between time to max slope, value of max slope, and AUC with varying viral concentrations applied to cell monolayers (**A**). Relationship between AUC and time to reach max slope for each variant. *p* value represents Spearman correlation (**B**).

Comparisons of slope characteristics reveal relationships between various aspects of the cell index curves. The input virus concentration does not appear to correspond with the value of max slope, except for Delta at 1 × 10^3^ to 1 × 10^5^ TCID_50_ dilutions. Relationships do exist between concentration and time to max slope, as well as concentration and AUC, with both time and AUC falling as viral input concentration increases (Figure 3A).

Time to max slope does correlate with AUC across all dilutions, with 1 × 10^4^ and 1 × 10^2^ TCID_50_ input concentrations having the highest correlations, at 0.9289 and 0.9132, respectively. All *p* values were below 0.0001, indicating a high degree of correlation (Figure 3B). The AUC and value of the max slope are somewhat related, with clustering occurring among variants. The exception to this is Delta, with only small clustering at the highest viral input, and very little among other dilutions (Figure S2). Time to max slope and value of max slope are somewhat similar overall to the AUC/max slope value relationship, with variants other than Delta clustering tightly together, with Delta showing weaker relationships between these variables, for unknown reasons (Figure S3).

### 3.4. Trends in Slope Characteristics Follow Visually Captured Cell Death

**Figure 4:**
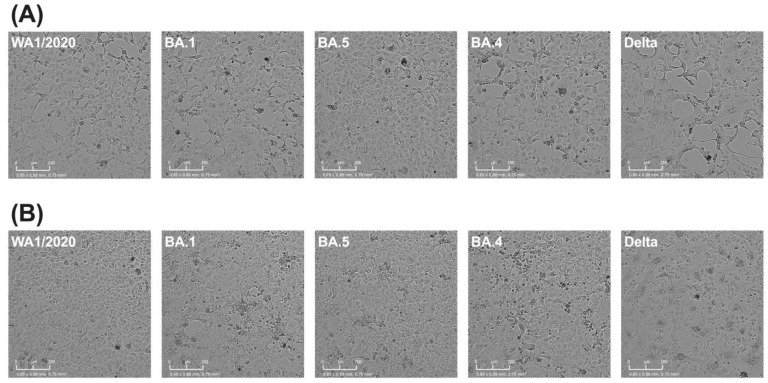
Monolayers visualized during replication. Images of monolayers taken 5 h prior to each variant’s maximum slope time point for Vero/TMPRSS2 inoculated with (**A**) 1 × 10^5^ TCID_50_ or (**B**) 1 × 10^4^ TCID_50_ SARS-CoV-2.

Images of each monolayer at the same spot in each well were captured at 60 min intervals over the course of the experimental period, allowing for generation of time lapse videos. This enables the cytopathic effect (CPE) to be interpreted visually during viral infection with each variant. At 1 × 10^5^ TCID_50_ input, WA1/2020 reaching widespread cytotoxicity can be seen before other variants, with BA.1 showing the slowest progression. The progression of Delta can be seen as a slow initial development of CPE, followed by a rapid progression across the monolayer once CPE begins. Overall, Delta is most similar to BA.4 and BA.5 visually, with WA1/2020 and BA.1 being the most rapid and most slow, respectively. This trend mirrors that seen in the slope characteristics (Videos S1–S5). Comparisons between variants taken at the same point, 5 h prior to the max slope timepoint, show a contrast between Delta and other variants. At concentrations of 1 × 10^4^ and 1 × 10^5^, the effect of cellular fusion appears evident from visual inspection of the monolayer (Figure 4). The effect is less apparent but still visible at lower concentration (Figure S4). Notably, these figures represent selectively chosen images but Videos S1–S5 are available for a more comprehensive assessment by readers.

## 4. Discussion

Here, we investigated differences in replication kinetics of multiple clinically relevant SARS-CoV-2 variants of concern using a new method termed real-time cell analysis (RTCA) with the Agilent xCELLigence eSight system, which measures electrical impedance of a cell monolayer in addition to acquiring live cell images using an onboard microscope and camera. Specifically, this approach approximates viral replication by assessing monolayer integrity and how it is altered by virus-induced cytolysis. An arbitrary unitless measure termed cell index is generated, allowing simultaneous quantification of multiple aspects of the cell index curves over a time course of monolayer infection with known viral input concentrations, including area under the curve (AUC), time to max slope, and the actual value of the max slope. This method is high throughput and allows kinetic measures of viral replication without the need to sample the assay.

Since this assay assesses cell death as a proxy for viral replication, it has several important caveats, and discoveries made using this assay should be validated by more traditional assays that directly measure viral infection and replication. In this report, we provide an initial assessment of variant-specific differences in viral replication in a single cell type. Our data were validated using a more traditional assay and were corroborated by published data on these variants. This assay also has several advantages over traditional assays. Most traditional assays require extensive hands-on time for sample collection at specific time points, potentially including inconvenient overnight timepoints, followed by downstream processing for PCR or antibody labeling. In contrast, the assay we describe herein requires only initial setup followed by data analysis, as data are collected automatically over time for the entirety of the preplanned assay. Thus, this assay greatly reduces the use of personal protective equipment (PPE) required for entry and work in high-containment labs and eliminates the costs associated with downstream wet lab procedures.

This approach highlighted several differences between variants that may be clinically relevant, most of which have been observed using more classical assays, validating our methods. We found that WA1/2020 reached max slope more quickly than other variants, indicating that it reaches a point during replication of cellular destruction more quickly than others. BA.5 was the closest to this rapid pace, and BA.1 was the slowest. Timelapse images of each variant concur with measures of cell index and allow visualization of virus-induced cytolysis. These data suggest that mutations accumulated or lost in BA.5 relative to BA.1 explain this kinetic difference and may indicate selection to regain a greater level of replicative ability lost in the BA.1 variant. Whether these changes have any impact on pathogenicity is difficult to address. Indeed, if any of the newly emerging subvariants of Omicron have recapitulated the pathogenicity of earlier, pre-Omicron variants, this effect might be masked in humans by the high global incidence of prior vaccination or infection, particularly with the BA.1 variant, which has led to very high levels of global immunity, which undoubtedly reduces disease and death associated with subsequent infection with any variant. Thus, any increase in pathogenicity associated with specific variants should be rigorously addressed in animal models.

The absolute value of the slope correlates to how quickly the monolayer loses its ability to resist current, indicating viral destruction of the monolayer. Delta was the highest variant, by far, for this metric, indicating that it rapidly destroys the monolayer, despite its slight delay reaching that slope as compared to WA1/2020. As before, BA.1 was the slowest here, indicating a slower replication potential. It is intriguing to speculate what might explain Delta’s uniqueness in this measure. Other studies have indicated that Delta replicates faster than other variants using different measures than we used here [47], but those measures may fail to identify the nuanced difference between Delta and the ancestral WA1/2020 that RTCA captures in our assays. When Delta was initially detected, there was much research into identifying mutations that might have led to its increased replicative capacity and possible pathogenicity. One mutation in particular, spike P681R, was shown to increase both fusogenicity and pathogenicity in hamsters [48,49]. Images acquired in our assays confirm the increased fusogenicity of this variant and provide a means to observe this phenomenon kinetically.

Taken together, our data reveal or confirm several aspects of viral replication that may shed light on the ongoing COVID-19 pandemic. First, we found that the Omicron subvariant BA.5 recapitulates most replicative features of the ancestral WA1/2020 variant, suggesting selection to regain features missing in the BA.1 variant. Second, our data reveal a unique replication profile for the Delta variant, which has been shown to induce greater pathogenicity in animal models and possibly in humans as well. Finally, our data add to a large body of work comparing replication profiles of viral variants. Many of these reports have found that the original Omicron variant, now termed BA.1, was demonstrably and significantly less fit in terms of replicative ability than any other tested variant, which may very well correlate with the clearly reduced pathogenicity of this variant. The fact that this variant swept the globe, infecting far more people than all other variants, possibly suggests that its immense immune evasion capacity far outweighed its reduced replicative ability, allowing for widespread infection including in those with pre-existing immunity. However, kinetic differences between specific variants are surprisingly complex. Previous reports showed that Omicron showed greater replication than Delta in nasal cells and bronchi explants while the opposite is true in lung explants and lung cells [35,36,37,38]. Underlying these differences are differential reliance on TMPRSS2 for entry [39], with Omicron relying much less on TMPRSS2 for cell entry than Delta. Infection with the original Omicron variant (BA.1) was associated with greatly reduced case fatality ratios for reasons that remain unknown. In vitro replication profiles may shed light on this clinically important observation. Thus, our data contribute to existing literature on these potentially important differences, and with several caveats and advantages discussed above.

Data represented here are a first step toward using this approach specifically to quantify differential viral kinetics of variants of SARS-CoV-2. Important future directions would be to test multiple cell types, provided that infection of those cells can be detected either via cell death (as measured by changes in impedance, or cell index) or directly observable changes in cellular phenotype, such as the formation of syncytia. Notably, this instrument can also image and quantify production of fluorescent molecules, such as GFP, in live cells. Thus, replication of recombinant viruses that express GFP during viral replication could also be assessed and quantitatively compared. Other modifications might include the addition of viral inhibitors such as interferons, antibodies, or pharmaceuticals.

Together, the work described here further elucidates the patterns of replication exhibited by each variant of SARS-CoV-2, with added clarity of real-time cell analysis allowing us greater insight into potential replication kinetics across time points not typically examined. Real-time cell analysis is a robust method that, in conjunction with established tools including qPCR, genomics, animal modeling, and public health surveillance, will give us greater insight into the unique nature of newly emerging variants.

## Data Availability

Data is contained within the article or supplementary materials.

## Supplementary Materials

The following supporting information can be downloaded at: https://www.mdpi.com/article/10.3390/v15091937/s1, Figure S1: Mean cell index over time of Vero/TMPRSS2 monolayers inoculated with SARS-CoV-2 variants; Figure S2: Comparisons of AUC and max slope values; Figure S3: Comparisons of max slope value and time to max slope; Figure S4: Monolayers visualized during replication; Video S1: Timelapse of monolayer destruction by the WA1/2020 variant; Video S2: Timelapse of monolayer destruction by the Delta variant; Video S3: Timelapse of monolayer destruction by the BA.1 variant; Video S4: Timelapse of monolayer destruction by the BA.5 variant; Video S5: Timelapse of monolayer destruction by the BA.4 variant.
