# Supplementary material for: Real-Time Analysis of SARS-CoV-2-Induced Cytolysis Reveals Distinct Variant-Specific Replication Profiles
Source: Viruses. 2023 Sep 16;15(9):1937. doi: 10.3390/v15091937 (PMC10537736; doi:10.3390/v15091937)
Supplement: Supplementary file 1 [file viruses-15-01937-s001.zip › viruses-2523672-supplementary materials.pdf]

Figure. S1. Mean Cell Index over time of Vero/TMPRSS2 monolayers inoculated with SARS- CoV-2 Variants.

Cell index was determined every 15 minutes over the course of 5 days. Data was normalized at 11.756h. Legend indicates quantity of virus in TCID50 added onto cell monolayers.

Figure. S2. Comparisons of AUC and Max Slope Value

Relationship between AUC and absolute value of max slope for each variant.

Figure. S3. Comparisons of Max Slope Value and Time to Max Slope

Relationship between absolute value of max slope and time to max slope for each variant at different $TCID_{50}$ values.

Figure. S4. Monolayers visualized during replication

Images of monolayers taken 5 hours prior to each variant's maximum slope time point for Vero/ TMPRSS2 inoculated with (A) $1x10^3$ $TCID_{50}$ or (B) $1x10^2$ $TCID_{50}$ SARS-CoV-2.

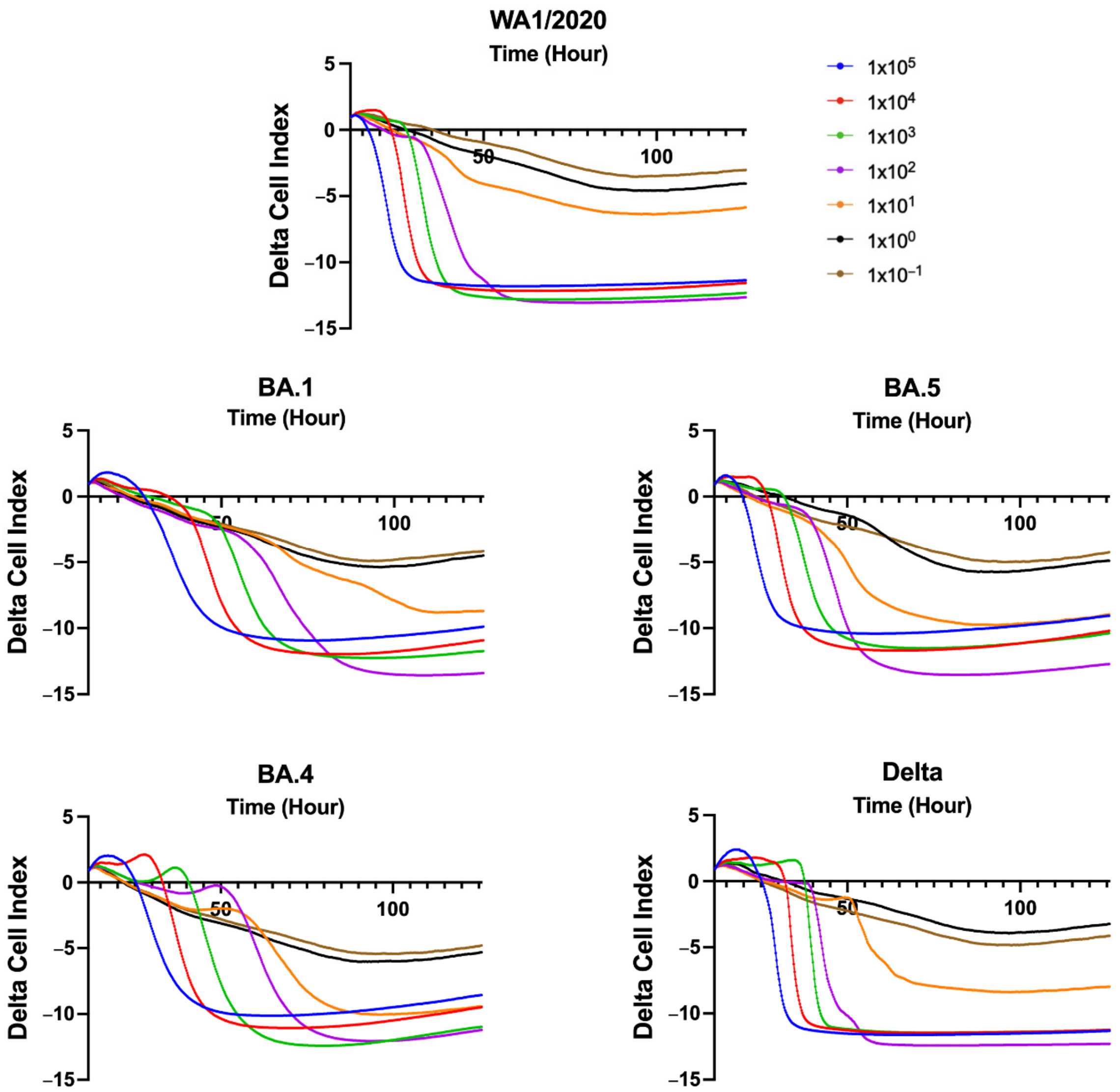
WA1/2020
Time (Hour)
Delta Cell Index
5
0
−5
−10
−15
50
100
1x10^5
1x10^4
1x10^3
1x10^2
1x10^1
1x10^0
1x10^−1
BA.1
Time (Hour)
Delta Cell Index
BA.5
Time (Hour)
Delta Cell Index
BA.4
Time (Hour)
Delta Cell Index
Delta
Time (Hour)
Delta Cell Index

Figure. S1

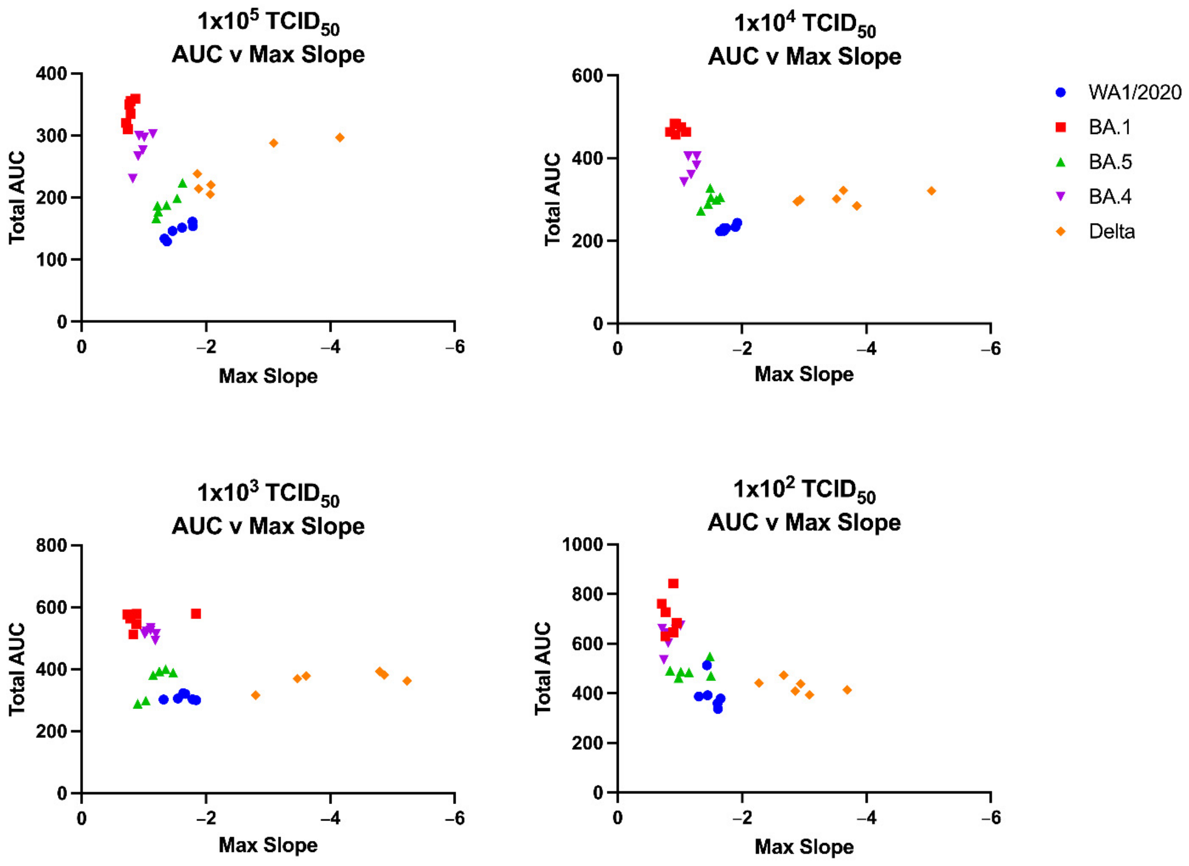

$1x10^5$ $TCID_{50}$
AUC v Max Slope
$1x10^4$ $TCID_{50}$
AUC v Max Slope
$1x10^3$ $TCID_{50}$
AUC v Max Slope
$1x10^2$ $TCID_{50}$
AUC v Max Slope
Total AUC
Max Slope
WA1/2020
BA.1
BA.5
BA.4
Delta

Figure. S2

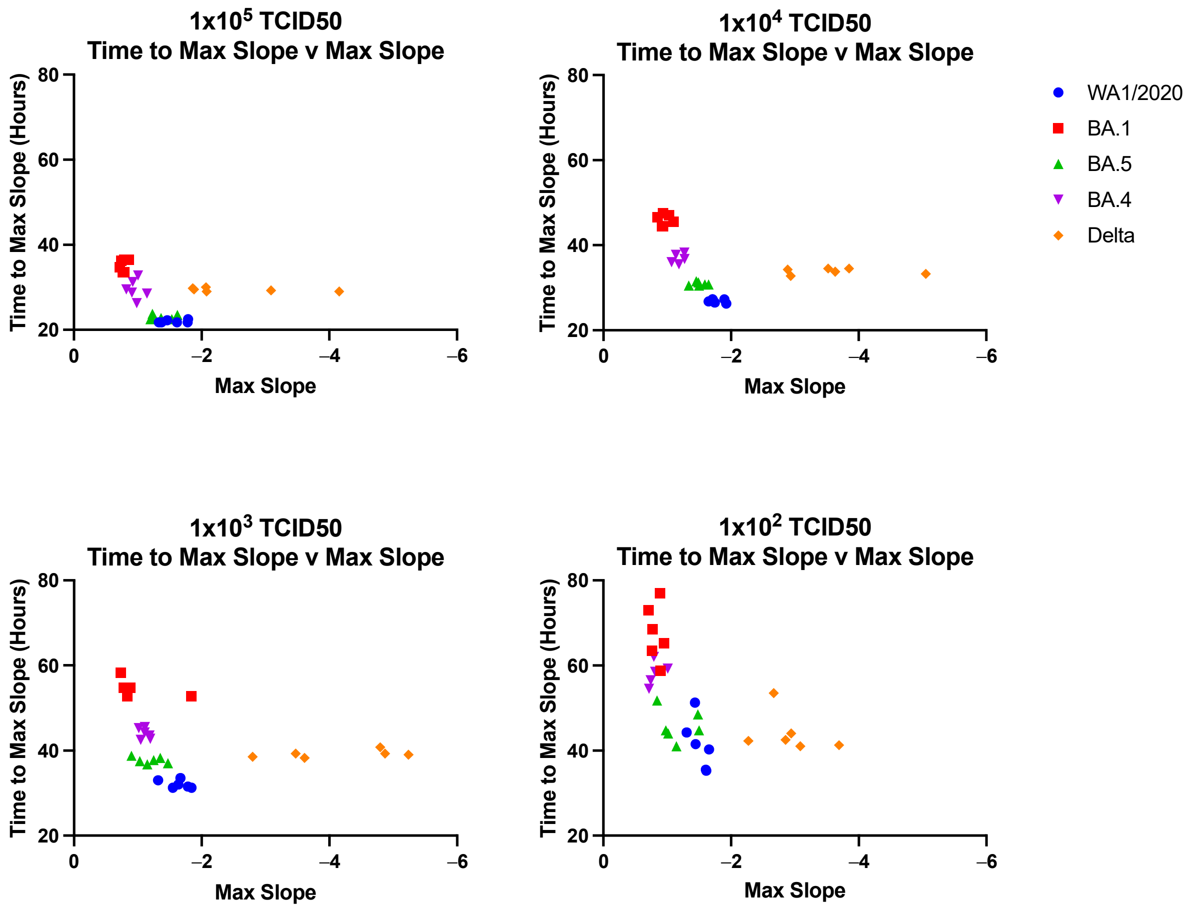
1x10^5 TCID50
Time to Max Slope v Max Slope
1x10^4 TCID50
Time to Max Slope v Max Slope
1x10^3 TCID50
Time to Max Slope v Max Slope
1x10^2 TCID50
Time to Max Slope v Max Slope
Time to Max Slope (Hours)
Max Slope
WA1/2020
BA.1
BA.5
BA.4
Delta

Figure. S3

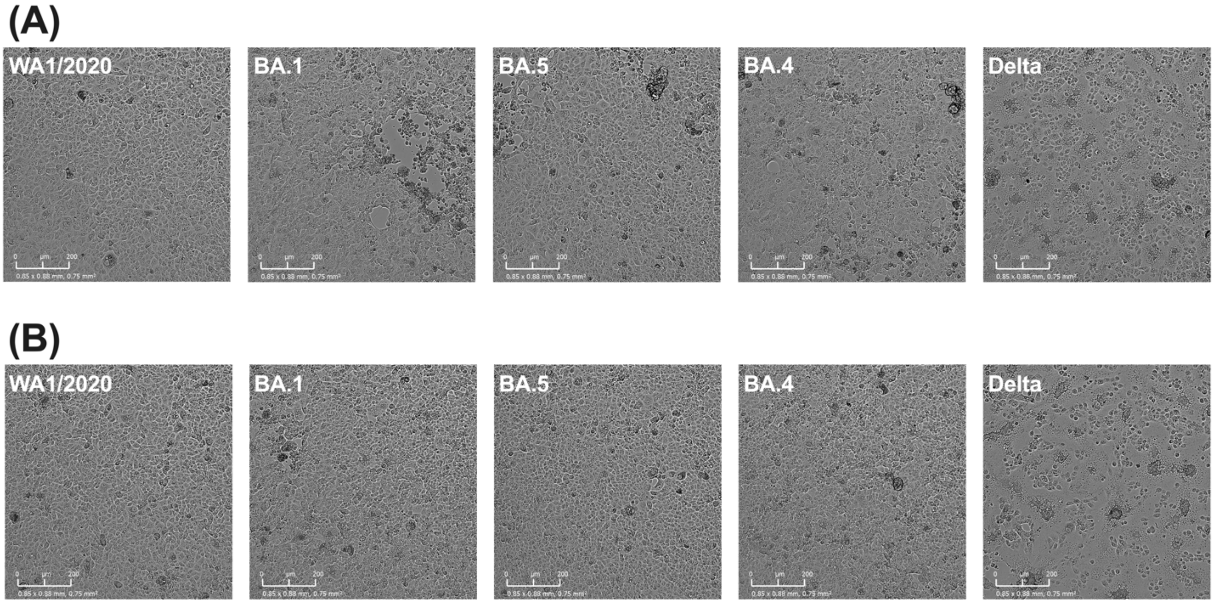

Figure. S4
